# Evaluation of the 12-Gene Molecular Score and the 21-Gene Recurrence Score as Predictors of Response to Neo-adjuvant Chemotherapy in Estrogen Receptor-Positive, HER2-Negative Breast Cancer

**DOI:** 10.1245/s10434-019-08039-7

**Published:** 2020-01-06

**Authors:** Hatem Soliman, Susanne Wagner, Darl D. Flake, Mark Robson, Lee Schwartzberg, Priyanka Sharma, Anthony Magliocco, Ralf Kronenwett, Johnathan M. Lancaster, Jerry S. Lanchbury, Alexander Gutin, William Gradishar

**Affiliations:** 1 Moffitt Cancer Center, Tampa, FL USA; 2 Myriad Genetics, Inc., Salt Lake City, UT USA; 3 Memorial Sloan Kettering Cancer Center, New York, NY USA; 4 Division of Hematology/Oncology, The University of Tennessee Health Science Center, West Cancer Center, Memphis, TN USA; 5 University of Kansas Medical Center, Kansas City, KS USA; 6 Myriad International Gmbh, Cologne, Germany; 7 Northwestern University, 676 N St. Clair, Suite 850, Chicago, IL 60611 USA

## Abstract

**Background:**

Neo-adjuvant chemotherapy (NaCT) facilitates complete surgical resection in locally advanced breast cancer. Due to its association with improved outcome, complete pathologic response (pCR) to neo-adjuvant treatment has been accepted as a surrogate for long-term outcome in clinical trials of human epidermal growth factor receptor 2 (HER2)-positive, triple-negative, or luminal B breast cancer patients. In contrast, NaCT is effective in only ~ 7–10% of estrogen receptor (ER)-positive, HER2-negative disease. Response biomarkers would enable such patients to be selected for NaCT.

**Methods:**

Two commercially available breast cancer prognostic signatures [12-gene molecular score (MS) and the 21-gene Recurrence Score (RS)] were compared in their ability to predict pCR to NaCT in ER-positive, HER2-negative breast cancer in six public RNA expression microarray data sets. Scores were approximated according to published algorithms and analyzed by logistic regression.

**Results:**

Expression data were available for 764 ER-positive, HER2-negative breast cancer samples, including 59 patients with pCR. The two scores were well correlated. Either score was a significant predictor of pCR (12-gene MS *p* = 9.4 × 10^−5^; 21-gene RS *p* = 0.0041). However, in a model containing both scores, the 12-gene MS remained significant (*p* = 0.0079), while the 21-gene RS did not (*p* = 0.79).

**Conclusions:**

In this microarray study, two commercial breast cancer prognostic scores were significant predictors of response to NaCT. In direct comparison, the 12-gene MS outperformed the 21-gene RS as a predictive marker for NaCT. Considering pCR as surrogate for improved survival, these results support the ability of both scores to predict chemotherapy sensitivity.

**Electronic supplementary material:**

The online version of this article (10.1245/s10434-019-08039-7) contains supplementary material, which is available to authorized users.

Over the last decade preoperative systemic therapy has become standard treatment for locally advanced breast cancer and is a treatment option for many patients with early-stage breast cancer.^1^ Neo-adjuvant chemotherapy (NaCT) frequently results in down-staging of both primary tumor and local nodes, rendering previously inoperable tumors amenable to surgical resection. Complete pathological response (pCR) to NaCT is a surrogate marker for improved survival of patients with human epidermal growth factor 2 (HER2)-positive, triple-negative, or luminal B breast cancer.^2^ In addition, neo-adjuvant treatment enables assessment of sensitivity to specific drugs in vivo and allows for response-guided therapeutic strategies. On the other hand, neo-adjuvant treatment delays resection of the primary tumor and carries the risk of tumor cell dissemination during the treatment interval. In particular for patients who are non-responsive to NaCT, the risk–benefit ratio may be unfavorable. While estrogen receptor (ER)-negative breast tumors show substantial rates of pCR, complete response to NaCT in ER-positive breast tumors is limited.^3^ Patients with ER-positive breast cancer thus would benefit most from predictive markers that would allow enrichment of patients with potentially responsive disease and avoidance of ineffective treatment for others.

Breast tumor RNA expression profiles have been highly successful as prognostic markers in the post-surgical setting.^4^–^8^ A number of RNA expression signatures from formalin-fixed, paraffin-embedded (FFPE) surgical specimens are now commercially available to assess prognosis in early stage, ER-positive breast cancer. Clinical guidelines have incorporated the use of such assays for the purpose of selecting patients with early stage, ER-positive disease for post-surgical chemotherapy.^1^^,^^9^ Each expression score defines patients of low, intermediate, and high risk of recurrence based on the expression of a variable number of genes that consistently include a module measuring proliferation. Several studies have shown that high-risk estimates in breast cancer prognostic profiles are driven primarily by high expression of the proliferation module.^10^^,^^11^

The 21-gene Recurrence Score (RS) and the 70-gene assay (MammaPrint) were the first prognostic assays to achieve clinical acceptance and were validated in prospective randomized trials.^12^^,^^13^ They have been followed by several “second generation” expression signatures [Breast Cancer Index (BCI), 12-gene Molecular Score (MS; EndoPredict), PAM50 risk of recurrence score (Prosigna)].^5^^,^^6^^,^^8^ Retrospective analyses in the translational arm of the ATAC trial have allowed direct comparison of four commercial breast cancer prognostic tests, with second generation expression scores generally outperforming the 21-gene RS as prognostic markers.^14^–^16^

Numerous studies have evaluated individual breast prognostic profiles for their ability to predict response to NaCT and, in some cases, neo-adjuvant endocrine therapy.^17^–^26^ However, little is known of their comparative value in predicting neo-adjuvant response. Here we use six public microarray expression data sets and approximations of one second-generation signature, the 12-gene MS, as well as one first generation signature, the 21-gene RS, to compare their ability to predict response to NaCT from pre-treatment breast tumor biopsies.

## Methods

### Data Sets

Public microarray gene expression data were obtained from the Gene Expression Omnibus (GEO) database.^27^ Data sets were selected if patients had been diagnosed with breast cancer, received NaCT, included at least 50 patients with ER-positive, HER2-negative disease, had expression data from pre-treatment fresh-frozen biopsies, made available treatment-associated outcome data on pathological response, and had been analyzed on commercial microarrays. Six data sets met all criteria: GEO accessions GSE20194, GSE20271, GSE25066, GSE32646, GSE34138, and GSE41998.^28^–^33^ Five studies used Affymetrix Human Genome U133 arrays, one study employed Illumina WG6 v3 bead chips (Table 1). Data from Affymetrix arrays were downloaded as.CEL files and normalized using RMA. Illumina data were between-array normalized by simple scaling (SSN). As all data were from public microarray datasets, oversight by an institutional review board was not required.

**Table 1:** Overview of microarray data sets used in this study

GSE#	Patients (n)	pCR (n)	ER +/HER2- patients (n)	pCR (n)	NaCT	pCR definition	Array type	References
20271	178	26	89	6	FAC, T/FAC^a^	No residual invasive disease in the breast or lymph nodes	Affymetrix Human Genome U133A Array	[27]
20194	278	56	140	7	T/FAC^a^	No residual invasive cancer in the breast or lymph nodes	Affymetrix Human Genome U133A Array	[26]
32646	115	27	55	5	P/FEC^b^	No evidence of residual invasive cancer in both breast and axilla	Affymetrix Human Genome U133 Plus 2.0 Array	[29]
41998	279	69	93	10	AC + P, AC + I^c^	No evidence of residual invasive adenocarcinoma in the breast and axillary lymph nodes	Affymetrix Human Genome U133A 2.0 Array	[31]
25066	486	91	268	27	Taxane + anthracycline-based CT	No residual invasive disease in the breast or lymph nodes	Affymetrix Human Genome U133A Array	[28]
41656	178	28	119	4	AC, FAC, T/FAC, T/FEC, T, D, DC^d^	Complete absence of invasive tumor cells in the breast and lymph nodes	Illumina HumanWG-6 v3 beadchip	[30]
Total	1514	297	764	59				

^a^F = fluorouracil, A = doxorubicin, C = cyclophosphamide, T = paclitaxel

^b^P = paclitaxel, F = fluorouracil, E = epirubicin, C = cyclophosphamide

^c^A = doxorubicin, C = cyclophosphamide, P = paclitaxel, I = ixabepilone

^d^F = fluorouracil, A = adriamycin, C = cyclophosphamide, T = taxol, E = epirubicin, D = doxetaxel, C = celecoxib

### Derivation of the 12-Gene Molecular Score

The 12-gene MS contains four normalization and control genes and eight target genes linked to proliferation and the ER pathway. Expression probes for the eight target genes (*AZGP1*, *BIRC5*, *DHCR7*, *IL6ST*, *MGP*, *RBBP8*, *STC2*, *UBE2C*) were averaged by gene, and gene averages combined into a 12-gene MS according to the published algorithm.^6^ At least one probe was available for all genes in all data sets.

### Derivation of the 21-Gene Recurrence Score

Sixteen genes in the 21-gene RS are prognostic markers with the remaining five housekeeper genes being used for normalization. Expression probes for the 16 target genes (*AURKA*, *BAG1*, *BCL2*, *BIRC5*, *CCNB1*, *CD68*, *CTSL2*, *ERBB2*, *ESR1*, *GRB7*, *GSTM1*, *MKI67*, *MMP11*, *MYBL2*, *PGR*, *SCUBE2*) were averaged by gene, and gene averages combined into gene groups for proliferation, ER, HER2 and invasion scores as described.^4^ At least one probe per gene was available on all arrays with the exception of *MYBL2* in GSE34138. A threshold was applied to the proliferation score based on the 80th percentile in ER-positive patients (Supplementary Material).^34^–^36^ Gene group averages and single gene expression values were combined into a 21-gene RS as published.^4^

### Association with Response

ER-positive, HER2-negative samples were selected based on immunohistochemistry (IHC) or fluorescent in situ hybridization (FISH) status in the accompanying clinical file. For each data set the two expression scores were transformed into *z*-scores and used as continuous variables. The score ranges differ from those observed on clinical reports from each score due to the different measurement platform and the z-score transformation. Response was defined as pCR obtained from the clinical data. pCR was determined by histological examination of the surgical sample and defined in a similar way across data sets as no residual invasive disease in the breast and lymph nodes. Association of each score with response was tested by logistic regression. To adjust for differences by data set, the cohort name was added as a categorical variable. In the combined analysis the model included both scores and adjustment for cohort. All analysis was performed in R 3.5.0 (R Foundation, 2018).

## Results

Of the 1514 patient samples in the six data sets, 782 qualified as ER-positive, HER2-negative by IHC and/or FISH status. Response data were complete for 764 patients with ER-positive, HER2-negative tumors. This included 59 complete pathological responses (8% response rate, Table 1).

### Correlation Between Expression Scores

The microarray-approximated 12-gene MS and 21-gene RS were moderately well correlated (*r* = 0.71) with correlation coefficients similar to those seen in quantitative PCR data.^37^ Correlations between scores (Supplementary Figure 1) as well as distributions of each score (Supplementary Figure 2) were similar across data sets.

### Association with Neo-adjuvant Response

**Fig. 1:**
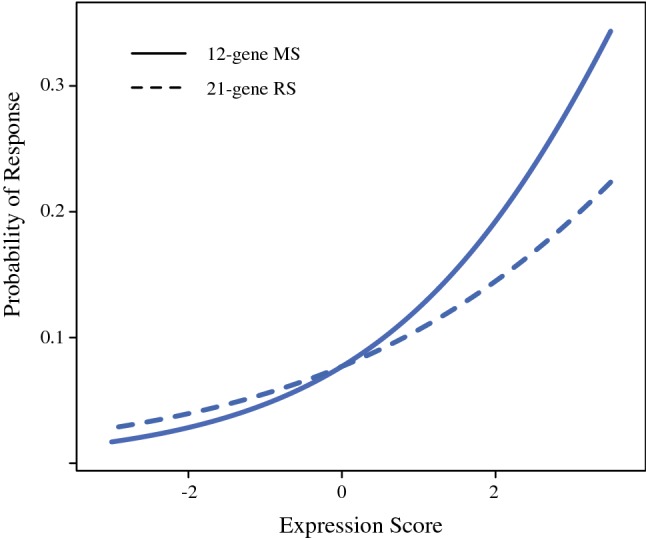
Probability of response to NaCT predicted by the 12-gene MS or the 21-gene RS

Each expression score was tested for its ability to predict pCR after NaCT in logistic regression models adjusted for cohort. When analyzed separately, either score was predictive of pCR (12-gene MS *p* = 9.4 × 10^−5^, 21-gene RS *p* = 0.0041; Table 2) with higher expression scores indicative of increased probability of response. Probability of response as a function of either expression score is depicted in Fig. 1. The probability of response for the 21-gene RS is higher at low scores and lower at high scores compared to the 12-gene MS; this indicates that the 21-gene RS score does not discriminate probability of response as well as the 12-gene MS. Additionally, when analyzed together in the same model, the 12-gene MS remained a significant predictor of response (*p* = 0.0079) while the 21-gene RS did not (*p* = 0.79), indicating that the 12-gene MS has additional discriminatory power not present in the 21-gene RS.

**Table 2:** Association of expression with NaCT response

Signature	OR	95% CI	*p*-value
Single score analysis^a^			
12-gene MS	1.69	1.30, 2.21	9.4 × 10^−5^
21-gene RS	1.42	1.12, 1.80	0.0041
Combined analysis^a^			
12-gene MS	1.63	1.14, 2.37	0.0079
21-gene RS	1.05	0.73, 1.46	0.79

^a^Logistic regression adjusted for cohort

Both the 12-gene MS and the 21-gene RS contain a subset of proliferation genes that are strongly weighted in the respective overall algorithm. However, only the 21-gene RS adds a threshold to the proliferation component.^4^ To test whether the percentile threshold employed affected the predictive power of the 21-gene RS score we performed a sensitivity analysis. The threshold for the proliferation group was varied from the 75th to the 90th percentile, a range guided by proliferation gene group expression distributions presented in previous publications.^34^–^36^ The 12-gene MS remained superior to the 21-gene RS in the combined analysis, irrespective of the proliferation group threshold (Supplementary Table 1). While variation in the proliferation threshold did not change the results of the combined analysis, the analyses of the individual score showed increasing loss of discriminatory power of the 21-gene RS with higher proliferation group thresholds.

## Discussion

NaCT is an effective tool for down-staging early and locally advanced breast cancer, but the sensitivity to NaCT varies greatly. Clinical parameters such as tumor size and node status have limited utility as response predictors.^29^^,^^30^^,^^38^ The most effective marker is ER status. Both in the neo-adjuvant and adjuvant systemic chemotherapy setting, ER-negative breast cancers show better complete response rates and higher absolute chemotherapy benefit, respectively compared to ER-positive tumors.^3^^,^^39^ ER-negative tumors are more highly proliferative and may therefore be particularly sensitive to agents that target DNA replication and/or cell division, such as NaCT. Similarly, luminal B tumors are more proliferative than the luminal A subtype and luminal B tumors have better response rates to NaCT.^20^ Breast cancer prognostic assays rely in large part on measurement of proliferation gene expression to discriminate risk and response to treatment. It is therefore not surprising that many breast cancer prognostic tests have been shown to predict NaCT.

We have compared the power of two breast cancer prognostic scores to predict NaCT response by using approximations of each score from microarray data. Using probe expression values for the published set of genes we employed the respective algorithms to create a 12-gene MS and 21-gene RS from pre-treatment samples in six NaCT treated breast cancer cohorts. As expected, both scores were predictive of NaCT response, as has been shown in other cohorts.^17^^,^^21^ In direct comparison however, the approximated 12-gene MS was a superior predictor than the approximated 21-gene RS. While the superior prognostic power of the 12-gene MS compared to the 21-gene RS was shown in a head-to-head comparison in the TransATAC cohort,^11^ this is to our knowledge the first direct comparison of these two prognostic scores as predictive markers for neo NaCT response. The reason for the better predictive power of the 12-gene molecular score could be that this signature includes a strong ER signaling motive beside the proliferation motive. This reflects previous findings that highly proliferative tumors with low ER expression are most sensitive to NaCT.^40^ A head-to-head comparison in a neo-adjuvant clinical trial could provide further information on the best predictive marker.

Limitations of this study include the use of microarray-based expression data and the limited availability of clinical parameters. The dynamic range for microarray expression data is generally more limited than that for quantitative PCR, especially with the lower end of the expression spectrum being lost to background. However, many of the currently available breast cancer profiles, including the 12-gene MS and the 21-gene RS, were developed from microarray data and the genes selected should be reliably measurable on microarrays.^4^^,^^6^ In addition, expression scores rely heavily on gene sets with generally high expression values in breast tumor tissue, i.e., proliferation and estrogen inducible genes. The 21-gene RS in particular, which thresholds the proliferation gene group upwards of the third quartile of the expression distribution,^36^ should be minimally affected by lack of discrimination at the lower end of expression values. The correlation between the approximated microarray versions of the 12-gene MS and the 21-gene RS is equivalent to that observed when the two scores are compared using quantitative PCR data.^37^ This suggests that any effect due to the measurement platform did affect both scores similarly. The observation in the sensitivity analysis of the decreasing power of the 21-gene RS to predict NaCT response with higher proliferation gene group thresholds is intriguing. While the effect of the threshold on the prognostic ability of the 21-gene RS is unknown, it is possible that the better performance of the second-generation prognostic profiles in the TransATAC comparisons may be partially be due to their lack of a proliferation gene threshold, thus enhancing the effect size of proliferation in these algorithms.^14^–^16^

The lack of availability of extended clinical factors for most public microarray studies prevented the inclusion of clinical parameters such as nodal status and tumor size. However, clinical tumor size and clinical node status are less reliable than post-surgical staging and their utility for predicting NaCT response varies widely across studies.^8^^,^^29^^,^^30^^,^^38^^,^^41^ Tumor features such as receptor status and subtype are more consistent measures of response. Moreover, continuous scores such as the various, widely available prognostic signatures could further improve response prediction. This is particularly desirable for tumor types where new and alternate therapies are available. Accumulating evidence suggests that neo-adjuvant endocrine therapy may be an effective alternate treatment strategy for ER-positive, HER2-negative breast cancer patients.^42^

Early assessment of tumor response to therapy could limit patient exposure to ineffective treatment and improve tumor control by prioritizing treatment of patients with higher probability of response. Here we show that gene expression scores such as the 12-gene MS and the 21-gene RS are predictive of pCR to NaCT and could therefore inform pre-surgical treatment decisions. Furthermore, since achieving pCR is broadly considered as a surrogate for improved survival in HER2-positive, triple negative, and luminal B breast cancer, both scores might predict long-term benefit from chemotherapy.

## Electronic supplementary material

Below is the link to the electronic supplementary material.

Supplementary material 1 (DOCX 276 kb)
