# Supplementary material for: Evaluation of the 12-Gene Molecular Score and the 21-Gene Recurrence Score as Predictors of Response to Neo-adjuvant Chemotherapy in Estrogen Receptor-Positive, HER2-Negative Breast Cancer
Source: Ann Surg Oncol. 2020 Jan 6;27(3):765–71. doi: 10.1245/s10434-019-08039-7 (PMC7000508; doi:10.1245/s10434-019-08039-7)
Supplement: Supplementary file 1 — Supplementary material 1 (DOCX 276 kb) [file 10434_2019_8039_MOESM1_ESM.docx]

**Supplemental Material**

*Selection of samples by hormone receptor status*

Five data sets had dichotomous estrogen receptor status by IHC positivity. In the GSE34138 cohort estrogen receptor status was given as percent ER positivity by IHC. Samples binned as 0 percent ER positivity were considered ER negative. All samples binned into 10% ER positivity and higher were categorized as ER positive.

*Thresholding of the 21-gene RS proliferation group*

# In the clinical setting expression values for both the 12-gene MS and the 21-gene RS are measured by qPCR. The seminal validation study of the 21-gene RS provides details on the derivation of the score from individual gene expression data.^1^ In the first step a subset of the 13 target genes is combined into four gene subgroups. A threshold is applied to the proliferation subgroup as well as to the HER2 gene group. The HER2 threshold is set to categorize HER2- from HER2+ samples. Since the analysis presented here excluded HER2 positive samples based on IHC data, no further thresholding of the HER2 group was necessary or attempted. To transfer the proliferation gene group threshold from quantitative PCR data to microarray values, we reviewed published data on the distribution on the proliferation gene group expression. A comparison of paired surgical samples and biopsies in 21 patients with invasive breast cancer showed all samples below the threshold.^2^ From a study examining the distribution of the 21-gene RS and its components in ILC we derived a threshold value at the 86^th^ percentile of the proliferation group distribution in ductal ILC.^3^ A 2015 analysis of 394,031 patients analyzed for the 21-gene RS showed consistent expression distribution of the gene groups across age categories with a median expression value of 5.4 and more than 75% of patients in each age group receiving proliferation gene group values below the threshold. For the full cohort, the proliferation gene group threshold would mark the 90% percentile of the distribution.^4^ Since this is consistent with the threshold value derived from the ILC study we selected a conservative threshold at the 80^th^ percentile of the proliferation expression distribution, assuming normal distribution, for the main analysis. To evaluate the effect of proliferation threshold selection on the performance of the 21-gene score as predictor of neo-adjuvant response we varied the threshold from the 75^th^ to the 90^th^ percentile of the proliferation score, with boundaries chosen to reflect threshold variation within the range observed in publications on qPCR data.

References

1. Paik S, Shak S, Tang G, et al. A multigene assay to predict recurrence of tamoxifen-treated, node-negative breast cancer. *The New England journal of medicine.* 2004;351(27):2817-2826.

2. Stull TS, Goodwin MC, Anderson JM, et al. P3-06-05: Comparison of Oncotype DX® Recurrence Scores between Surgical and Core Biopsy Specimens in Breast Cancer Patients. *Cancer Res.* 2011;71(24 Supplement):P3-06-05.

3. Anderson JM, Yoshizawa C, Winer EP, et al. Abstract P3-10-15: Quantitative Gene Expression by RT-PCR in Classic and Variant Forms of Lobular Carcinoma in Estrogen Receptor Positive Invasive Breast Cancer. *Cancer Res.* 2010;70(24 Supplement):P3-10-15.

4. Swain SM, Nunes R, Yoshizawa C, Rothney M, Sing AP. Quantitative Gene Expression by Recurrence Score in ER-Positive Breast Cancer, by Age. *Adv Ther.* 2015;32(12):1222-1236.

Supplementary Figure 1. Correlations between 21-gene RA and 12-gene MS scores.


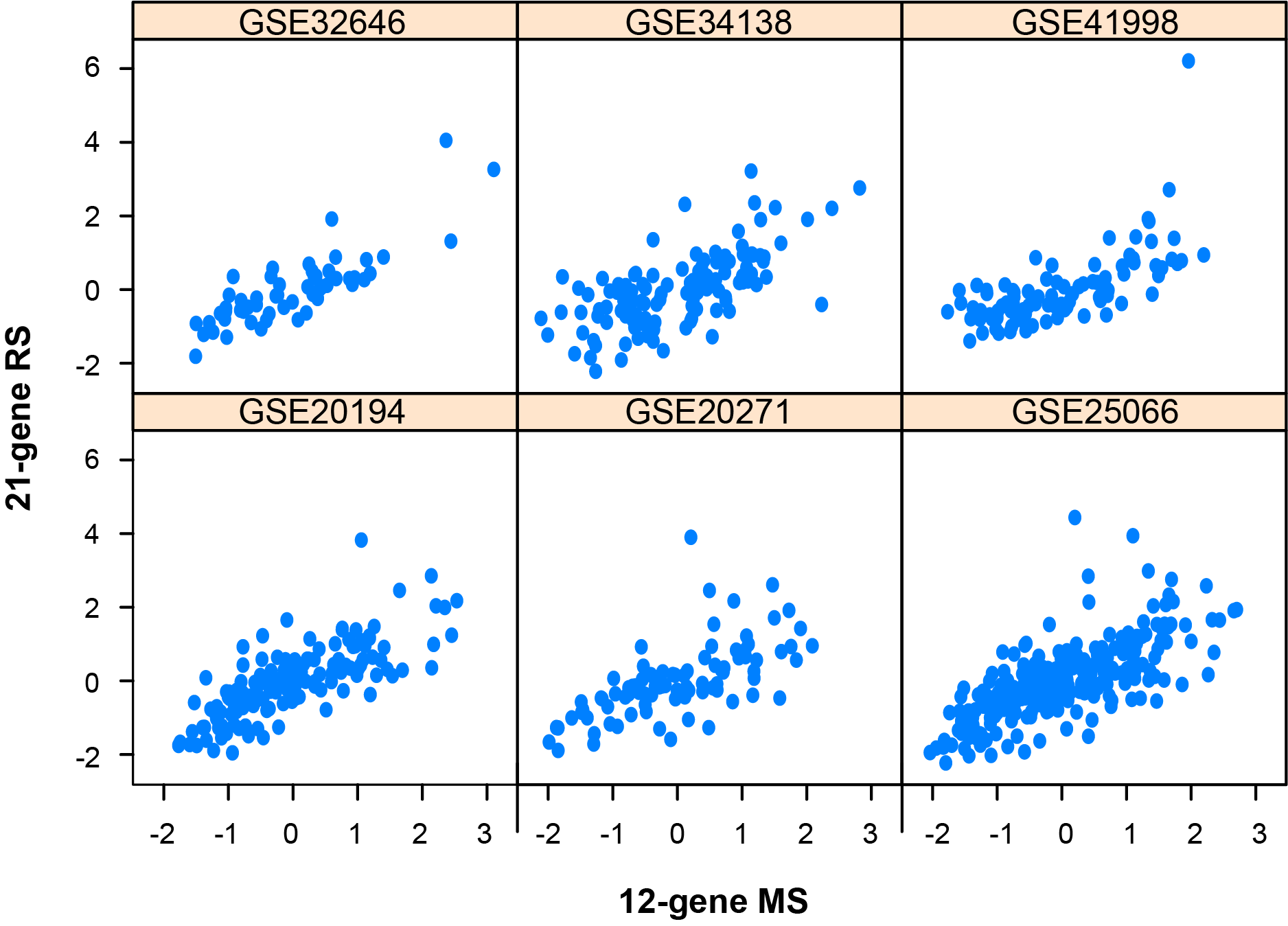


Supplementary Figure 2. Distributions of each score for both 12-gene MS and 21-gene RS.


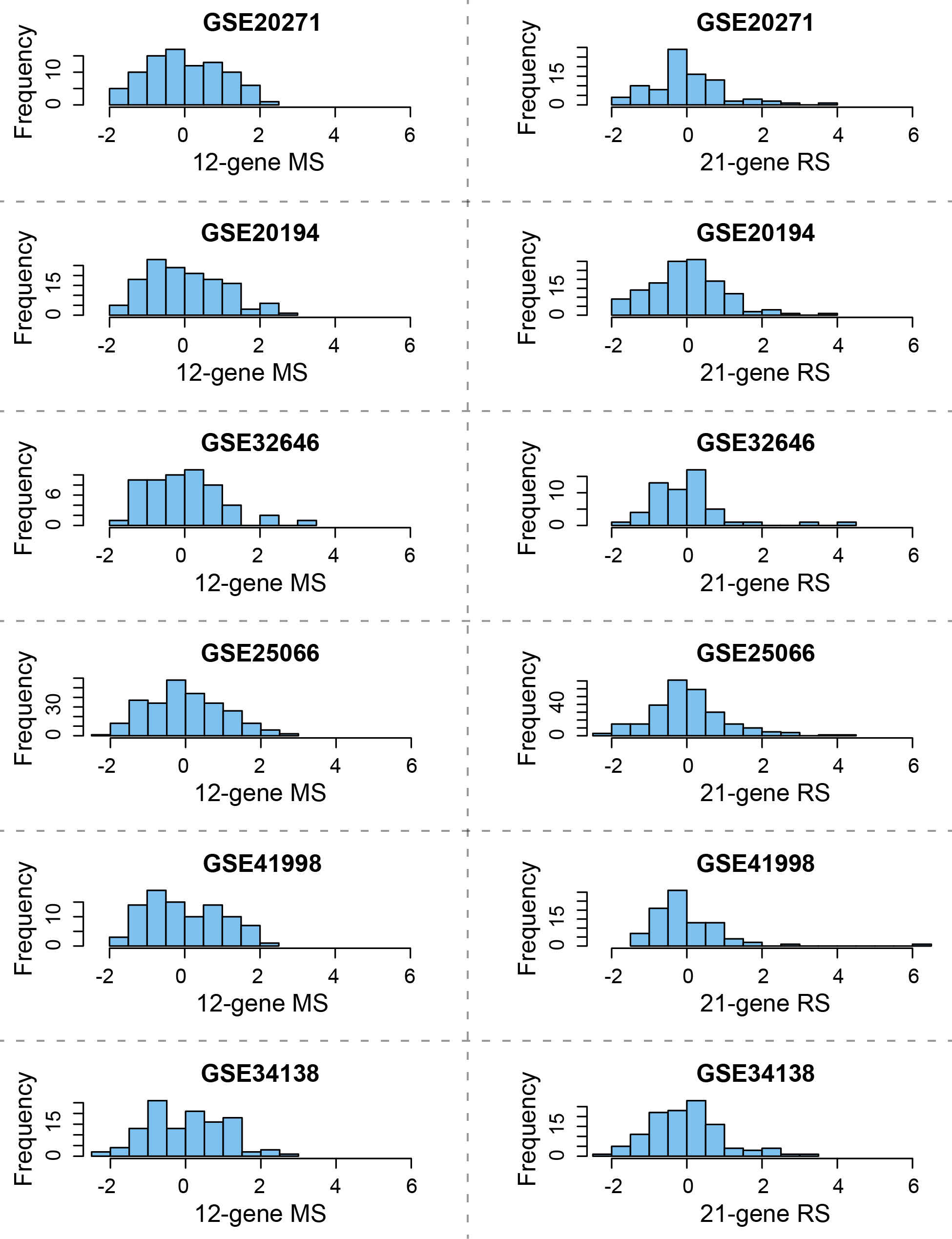


Supplementary Table 1. Comparison of 12-gene MS and 21-gene RS with different proliferation group thresholds.

| **Single Score Analysis** | | | | **Combined Analysis** | | | | **Threshold*** |
| --- | --- | --- | --- | --- | --- | --- | --- | --- |
| **12-gene MS** | | **21-gene RS** | | **12-gene MS** | | **21-gene RS** | |  |
| *OR (95% CI)* | *p-value* | *OR (95% CI)* | *p-value* | *OR (95% CI)* | *p-value* | *OR (95% CI)* | *p-value* |  |
| 1.69 (1.30, 2.21) | 0.000094 | 1.46 (1.15, 1.84) | 0.0022 | 1.59 (1.10, 2.33) | 0.014 | 1.08 (0.75, 1.51) | 0.66 | 75 |
|  |  | 1.42 (1.12, 1.80) | 0.0041 | 1.63 (1.14, 2.37) | 0.0079 | 1.05 (0.73, 1.46) | 0.79 | 80 |
|  |  | 1.40 (1.10, 1.76) | 0.0065 | 1.66 (1.16, 2.38) | 0.0050 | 1.03 (0.72, 1.42) | 0.88 | 85 |
|  |  | 1.37 (1.07, 1.72) | 0.012 | 1.69 (1.20, 2.39) | 0.0028 | 1.00 (0.71, 1.38) | 0.98 | 90 |

*percentile of proliferation gene group in the 21-gene RS in ER+ /HER2- patients
